# Cell-Free Expression and Photo-Crosslinking of the Human Neuropeptide Y_2_ Receptor

**DOI:** 10.3389/fphar.2019.00176

**Published:** 2019-03-01

**Authors:** Lisa Maria Kögler, Jan Stichel, Anette Kaiser, Annette G. Beck-Sickinger

**Affiliations:** Institute of Biochemistry, Faculty of Life Sciences, Leipzig University, Leipzig, Germany

**Keywords:** cell-free, human Y_2_ receptor, photo-crosslinking, G protein-coupled receptors, neuropeptide Y

## Abstract

G protein-coupled receptors (GPCRs) represent a large family of different proteins, which are involved in physiological processes throughout the entire body. Furthermore, they represent important drug targets. For rational drug design, it is important to get further insights into the binding mode of endogenous ligands as well as of therapeutic agents at the respective target receptors. However, structural investigations usually require homogenous, solubilized and functional receptors, which is still challenging. Cell-free expression methods have emerged in the last years and many different proteins are successfully expressed, including hydrophobic membrane proteins like GPCRs. In this work, an *Escherichia coli* based cell-free expression system was used to express the neuropeptide Y_2_ receptor (Y_2_R) for structural investigations. This GPCR was expressed in two different variants, a C-terminal enhanced green fluorescent fusion protein and a cysteine deficient variant. In order to obtain soluble receptors, the expression was performed in the presence of mild detergents, either Brij-35 or Brij-58, which led to high amounts of soluble receptor. Furthermore, the influence of temperature, pH value and additives on protein expression and solubilization was tested. For functional and structural investigations, the receptors were expressed at 37°C, pH 7.4 in the presence of 1 mM oxidized and 5 mM reduced glutathione. The expressed receptors were purified by ligand affinity chromatography and functionality of Y_2_R_cysteine_deficient was verified by a homogenous binding assay. Finally, photo-crosslinking studies were performed between cell-free expressed Y_2_R_cysteine_deficient and a neuropeptide Y (NPY) analog bearing the photoactive, unnatural amino acid *p*-benzoyl-phenylalanine at position 27 and biotin at position 22 for purification. After enzymatic digestion, fragments of crosslinked receptor were identified by mass spectrometry. Our findings demonstrate that, in contrast to Y_1_R, NPY position 27 remains flexible when bound to Y_2_R. These results are in agreement with the suggested binding mode of NPY at Y_2_R.

## Introduction

G protein-coupled receptors (GPCR) are involved in the regulation of processes throughout the whole body and are therefore potential pharmaceutical targets. In 2017, about 35% of all approved drugs used GPCRs as targets and still, there is huge potential left. With about 800 members, these proteins are the largest family of integral membrane proteins, and only 134 of them are targeted by pharmaceuticals by now (Sriram and Insel, 2018). In order to identify new drugs, new GPCR targets can be approached. Another concept is to find compounds for the fine-tuning of the GPCR signaling, which provides the opportunity to add other dimensions of regulation besides “on” or “off.” The rational design of novel drug compounds requires detailed knowledge of the binding pocket as well as the dynamic processes GPCRs undergo during activation. However, direct structural investigations of GPCRs remain challenging owing to their hydrophobicity and structural flexibility, since GPCRs adapt many distinct conformations in the basal, as well in the active state (Katritch et al., 2013; Venkatakrishnan et al., 2013; Manglik et al., 2015; Latorraca et al., 2017).

X-ray crystallography and NMR spectroscopy are commonly used for the investigations of proteins. Until 2017, crystal structures of more than 30 different GPCRs have been solved in different activation states as reviewed by Xiang et al. (2016) and Lee et al. (2018). These structures, solved in complex with agonists, antagonists, G proteins, arrestins or stabilizing proteins, give insights into the mechanisms underlying GPCR activation and their conformations in inactive or active states. A drawback of crystallization is that only one conformation at a time can be captured, giving a good state-of-the-art picture, but conformations with short half-life are often missed.

To fill this gap, mass spectrometry (MS) based structural investigations can be performed complementary to the classical protein structural methods. One approach is the combination of crosslinking strategies with MS, which can provide information about interaction partners or can be used to map interaction surfaces, as recently reviewed (Nguyen et al., 2018; Seidel and Coin, 2018; Sinz, 2018). In contrast to X-ray crystallography or NMR spectroscopy, only low quantities of protein are needed for the identification of crosslinking regions by MS. Furthermore, structural information is gained at physiological pH and reflects the native protein conformation in solution. Only minor limitations in the size of the target proteins are found for MS, because the cross-linked complexes are usually enzymatically digested before MS analysis.

With increasing application of crosslinking, more crosslinking agents are available, providing different reactivity and specificity. These include homobifunctional crosslinkers, “zero-length” chemical crosslinkers, MS cleavable crosslinker and photo-crosslinker (Sinz, 2006; Manglik et al., 2015; Hage et al., 2017; Sinz, 2017). The latter are chemically inert compounds, which, upon irradiation with UV light, form reactive intermediates that can react with adjacent molecules to form covalent bonds (Weber and Beck-Sickinger, 1997). Among the most frequently used photo-reactive groups are benzophenones moieties, phenyl- and alkyl-diazirines and phenyl-azides as reviewed several times (Preston and Wilson, 2013; Sumranjit and Chung, 2013; Smith and Collins, 2015). The benzophenone moiety can be site-specifically inserted into proteins and peptides by using Bpa, an unnatural amino acid, and several techniques including solid-phase peptide synthesis (SPPS) (Kauer et al., 1986), expansion of the genetic code (Chin et al., 2002, 2003; Hino et al., 2005) or semi-synthetic approaches (David and Beck-Sickinger, 2007). After UV irradiation, the benzophenone moiety is able to form a reactive triplet ketyl biradical, whose oxygen is able to abstract hydrogens from weak C-H-bonds in a hydrophobic environment resulting in ketyl and alkyl radicals. These radicals then combine to form a C–C bond (Preston and Wilson, 2013). One drawback of MS-based structural investigations is the need for high quality MS data, which means that the protein of interest must be available in a homogenous and functional form.

Different strategies have been developed to address this issue and most cross-linking approaches are performed with GPCRs expressed in eukaryotic cells. To obtain GPCRs in a more defined environment and in higher amounts recombinant expression can be performed in hosts like insect, mammalian or prokaryotic cells (Shukla et al., 2015). While the production in insect and mammalian hosts requires more complex growth media, the growth of the cells is slower and is in general more expensive. The expression in prokaryotic cells mostly leads to the accumulation of GPCRs in inclusion bodies. This requires time-consuming resolubilization, purification and refolding steps to achieve functional receptors. When considering photo-crosslinking with subsequent analysis by matrix-assisted laser desorption ionization time of flight (MALDI-ToF) MS it is additionally favorable to avoid post-translational modifications, which usually leads to more heterogeneous protein samples and further complicates the interpretation of the results.

An alternative approach for the production of soluble, homogenous GPCRs are cell-free (CF) expression systems. Due to their open nature, these systems allow a directed optimization of the expression conditions with only minor limitations toward the protein production itself. The expression can be completely guided toward the expression of the protein of interest, which includes the enhancement of expressed protein, as well as the co-translational addition of protein stabilizers like detergents or lipids (Bernhard and Tozawa, 2013) and the adjustment of the redox conditions (Bundy and Swartz, 2011; Michel and Wüthrich, 2012; Proverbio et al., 2013). Furthermore, CF approaches offer a high potential for the efficient incorporation of unnatural amino acids (Staunton et al., 2006; Bundy and Swartz, 2010; Hong et al., 2014), without limitations due to toxicity or lacking transport to the cell membrane.

The NPY receptor family consists of four rhodopsin-like GPCRs in humans (Pedragosa-Badia et al., 2013), which are potential targets for the treatment of diverse diseases including metabolic diseases (Yi et al., 2018), cardiovascular diseases (Tan et al., 2018), as well as certain cancer types (Li et al., 2015; Tilan and Kitlinska, 2016) and disorders of the central nervous system (Duarte-Neves et al., 2016; Gøtzsche and Woldbye, 2016; Reichmann and Holzer, 2016; Robinson and Thiele, 2017). The endogenous ligand NPY binds to the Y_1_R, the Y_2_R and the Y_5_R with high affinities, besides their relatively low sequence similarity (Brothers and Wahlestedt, 2010). It adopts diverse binding modes on the different NPY receptor subtypes, where it produces partially adverse effects. The binding mode of NPY to Y_2_R has been intensively studied by nuclear magnetic resonance (NMR) spectroscopy of recombinantly expressed and *in vitro* refolded receptor (Schmidt et al., 2009; Schmidt et al., 2017)in combination with mutagenesis studies and molecular modeling (Kaiser et al., 2015).

In this study we describe the soluble cell-free expression of two variants of the Y_2_R, C-terminally coupled to a fluorescent protein (Y_2_R_eGFP), as well as a cysteine minimized variant (Y_2_R_cysteine_deficient). We used an *Escherichia coli* (*E. coli*) based CF expression system and optimized it for receptor expression and solubilization. Active receptors were purified by ligand affinity chromatography. We furthermore performed a homogenous binding assay with the solubilized Y_2_R_cysteine_deficient to prove functionality. Photo-crosslinking studies were performed between Y_2_R_cysteine_deficient and an NPY analog, containing Bpa at position 27 and biotin at position 22, and the results were compared to the known binding mode of NPY(13–36) to Y_2_R (Kaiser et al., 2015).

## Materials and Methods

### Peptide Synthesis

Rink amide resin (4-(2,4-dimethoxyphenyl-Fmoc-aminomethyl)-phenoxy, 15 μmol scale, loading 0.69 mmol/g) was obtained from Iris Biotech (Marktredwitz, Germany), and NovaSyn TGR R resin (15 μmol scale, loading 0.18 mmol/g) from Merck KGaA (Darmstadt, Germany). 9-Fluorenylmethoxycarbonyl (Fmoc)- and *tert*-butyloxycarbonyl-protected amino acids were purchased from Orpegen OPC (Heidelberg, Germany), Iris Biotech, and Sigma-Aldrich (Taufkirchen, Germany). Orthogonally 1-(4,4-dimethyl-2,6-dioxocyclohexyl-idene)ethyl (Dde) protected amino acid Fmoc-Lys(Dde)-OH, as well as 6-(Fmoc-amino)-hexanoic acid (Ahx) and Fmoc-Bpa-OH, 1-[Bis(dimethyl-amino)methylene]-1*H*-1,2,3-triazolo[4,5-*b*]pyridi-nium 3-oxide hexafluorophosphate (HATU), 1-hydroxybenzotriazole (HOBt), ethyl2-cyano-2-(hydroxyimino)acetate (Oxyma), and *N,N′*-diisopropylcarbodiimide (DIC) were purchased from Iris Biotech. Biotin, *N,N*-diisopropylethylamine (DIPEA), hydrazine, piperidine, 1,2-ethanedithiol (EDT), thioanisole (TA), and trifluoroacetic acid (TFA) were obtained from Sigma-Aldrich and 5(6)-TAMRA Novabiochem^®^ from Merck KGaA. Acetonitrile (ACN) was from VWR (Darmstadt, Germany), dimethylformamide (DMF) and dichloromethane (DCM) were obtained from Biosolve (Valkenswaard, Netherlands), and *p*-thiocresol from Alfa Aesar (Ward Hill, MA, United States).

Porcine NPY (YPSKPDNPGEDAPAEDLARYYSALRHYINLITRQRY-NH_2_) and analogs were synthesized by a combination of automated and manual SPPS on an automated multiple peptide synthesis robot system (Syro, MultiSynTech, Bochum, Germany), using Fmoc/tBu strategy in 15 μmol scale as previously described (Pedragosa-Badia et al., 2014). The porcine variant of NPY, which contains a single exchange at position 17 (M17L), was used due to its increased solubility and stability. The porcine NPY possesses affinity and signaling properties identical to the human NPY. It will be following termed NPY. NPY, biotin-(Ahx)_2_-NPY, and 5(6)-TAMRA-Ahx(5-24)NPY were synthesized on Rink amide resin and [K^22^[(Ahx)_2_-biotin]Bpa^27^]NPY (Photo^27^-NPY) on a NovaSyn TGR R resin to obtain C-terminally amidated peptides.

All manual coupling reactions were performed at room temperature under constant shaking over night. For manual coupling of Ahx and Bpa 5 eq. of the respective Fmoc protected amino acid, 5 eq. HOBt and 5 eq. DIC in DMF was used. Biotin labeling was performed by dissolving 3 eq. biotin in DMF for 10 min at 60°C. Following, 3 eq. HOBt and 3 eq. DIC were added to the mixture, which was incubated on the resin. For 5(6)-TAMRA labeling 2 eq. 5(6)-TAMRA, 2 eq. HATU and 2 eq. DIPEA were dissolved in DMF and added to the respective resin.

Dde cleavage was obtained by addition of freshly prepared 2% (v/v) hydrazine in DMF for 10 min at least 10 times using an automated multiple peptide synthesis robot system.

Side chain deprotection and concurrent cleavage from the resin was accomplished by using a mixture of TFA/TA/EDT (90:7:3, 1 mL) or TFA/TA/TK (90:5:5 v/v/v) for Bpa containing peptides for 3 h at room temperature under constant shaking.

All peptides were purified by preparative reversed-phase high-performance liquid chromatography (RP-HPLC) on a PhenomenexKinetex 5u XB-C18 (5 μm, 100 Å), a PhenomenexAeris^TM^ Peptide XB-C18 (5 μm, 100 Å) or a Phenomenex Jupiter 10u Proteo C18 (90 Å, 10 μm) column using linear gradients of 0.08% (v/v) TFA in ACN (eluant B) in 0.1% (v/v) TFA in H_2_O (eluant A). Peptide purity was determined by analytical RP-HPLC using two different columns (PhenomenexAeris^®^ Peptide 3.6u XB-C18 (3.6 μm, 100 Å, 1.55 mL/min), PhenomenexKinetex 5u XB-C18, 5 μm, 100 Å, 1.55 mL/min). Identity was confirmed by matrix-assisted laser desorption ionization time of flight (MALDI-ToF) MS (Ultraflex III MALDI-ToF/ToF, Bruker Daltonics) and ESI-HCT MS (High-capacity ion trap ESI-MS, Bruker Daltonics). Purity of all peptides was ≥95%.

### Plasmids

Human Y_2_R_cysteine_deficient cDNA, containing the mutations C58^1.40^A, C103^2.57^S, C151^3.53^S, C272^6.39^A, C316^7.44^A, C342^7.70^A (Witte et al., 2013), was cloned into pVITRO2-hygro-mcs (InvivoGen, San Diego, CA, United States), and C-terminally fused to an enhanced yellow fluorescent protein (eYFP) by an ADPPVV linker containing GCGGATCCACCGGTCGTG on the DNA level. The chimeric G protein G_αΔ6qi4myr_ was cloned into pcDNA3 and was kindly provided by Kostenis (2001).

Human Y_2_ receptor cDNA fused to an enhanced green fluorescent protein (eGFP) by a GCGGATCCGGTGATGGTG linker (amino acid sequence ADPVMV) and human Y_2_R_cysteine_deficient cDNA were subcloned into the prokaryotic expression vector pET-22b(+) (Merck Millipore, Darmstadt, Germany) by using NdeI and XhoI (Thermo Fisher Scientific, Braunschweig, Germany). A C-terminal deca-histidine-tag (10 × His) linked by CTCGAG was added for purification and detection. The molecular weight of Y_2_R_eGFP_His_10_ is 71650.6 Da and the Y_2_R_cysteine_deficient has a molecular weight of 44184.4 Da.

### IP-One Assay

The inositol phosphate (IP)-one assay was performed as described previously (Wanka et al., 2018). In short, confluent HEK293 cells (human embryonic kidney) in 6-well plates were co-transfected with receptor and a chimeric G_α_ protein (G_αΔ6qi4myr_). On the next day the cells were trypsinated, re-seeded in white 384-well plates (Greiner Bio-one) and maintained over night at 37°C, 5% CO_2_ and 95% humidity (standard conditions). For measurement of IP production, the IP-one Gq assay kit (Cisbio Bioassay, Codolet, France) was used. The medium was removed and cells stimulated for 1.5 h with 15 μL stimulation solution containing increasing concentrations of NPY or NPY analogs. After cell lysis with lysis buffer supplemented with antibody 1 and antibody 2 according to the manufacturer’s protocol, the emission at 665 nm and 620 nm was measured in a Tecan Spark plate reader (Tecan Group AG, Männedorf, Switzerland).

Using GraphPad Prism 5.0 (GraphPad Software, San Diego, CA, United States) the concentration response curves were normalized to the NPY Y_2_R curve. The EC_50_ and *E*_max_ values of concentration response-curves were determined by non-linear regression (curve fit). The experiments were conducted in at least two independent experiments.

### [^125^I]PYY Binding Assay

For binding experiments, membrane preparations of transiently transfected HEK293 cells were used. For transfection, HEK293 cells were seeded into 75 cm^2^ culture flasks and were transiently transfected with pVitro2_Y_2_R_cysteine_deficient_eYFP (12 μg) with MetafectenePRO (Biontex Laboratories GmbH, Munich, Germany).

For membrane preparation, transiently transfected HEK293 cells were washed twice with Dulbecco’s phosphate-buffered saline (DPBS, Biochrome GmbH, Berlin, Germany) and afterwards harvested with DPBS. The cells were taken up in Tris-buffer [50 mM Tris, pH 7.5, with 1% (v/v) Pefabloc SC] and homogenized 50 times (25× loose and 25× tight pestle) with a manual dounce homogenizer and centrifuged for 10 min at 2,400 rpm, 4°C. The supernatant was centrifuged again for 30 min at 12,000 rpm and 4°C and the resulting pellet was resuspended in HEPES buffer [25 mM HEPES, 2 mM MgCl_2_ and 25 mM CaCl_2_ (Fluka, Buchs, Switzerland), pH 7.4], with 50 nM Pefabloc SC. The solution was homogenized as described above and centrifuged for 30 min at 12,000 rpm, 4°C. The resulting membrane pellet was thoroughly resuspended in HEPES-buffer without Pefabloc SC and protein concentration was measured in triplicates by Bio-Rad Protein Assay (Bio-Rad Laboratories, Inc., Hercules, CA, United States) against a BSA fraction V (BSA, Roth, Karlsruhe, Germany) standard. Next, 50 nM (final) Pefabloc SC was added and 100 μL aliquots were stored at -70°C.

The competition binding assay was previously described by Kaiser et al. (2018) with minor changes. Briefly, NPY, 5(6)-TAMRA-Ahx(5-24)NPY and [^125^I]-pPYY (NEX240, PerkinElmer, Boston, MA, United States) were diluted in water with 1% (w/v) BSA. 10 μl of increasing concentrations of NPY or 5(6)-TAMRA-Ahx(5-24)NPY, 10 μl of [^125^I]-PYY (60 pM final concentration) and 0.75 μg membrane preparations in 80 μL, diluted in HBSS with 1% (w/v) BSA, 50 nM Pefabloc SC to 0.75 μg total protein per well were added to a 96 well plate. The incubation was performed at room temperature and with continuous shaking for 3 h. The binding assay was terminated by filtering the samples though a glass fiber filter Printed Filtermat B (Perkin Elmer), which was presoaked with 0.1% polyethyleneimine (Fluka) in distilled water. The radioactivity was measured by using a FilterMate Harvester and Melt-on Scintillator Sheets MeltiLexA in a MicroBeta2 Plate Counter (2450 Microplate), purchased from PerkinElmer. Concentration-response curves were analyzed with GraphPad Prism 5.0, all curves were normalized to the top (100%) and bottom (0%) values of the NPY curve. The row means total function was used to summarize concentration-response curves of different experiments to one single concentration-response curve. IC_50_ and pIC_50_ ± s.e.m. were examined using logistic fitting with fixed Hill Slope (*n*_H_ = 1). Assays were performed in triplicates for at least two independent experiments. For *K*_d_ value determination of [^125^I]PYY Y_2_R_cysteine_deficient membrane preparations (5 μg total protein) were incubated with increasing concentrations of [^125^I]PYY in an overall volume of 250 μL for 3 h at room temperature under constant shaking. The assay was terminated by filtration and radioactivity measured in a liquid scintillation counter. Concentration-response curves were analyzed with GraphPad Prism 5.0 and *K*_d_ values were determined by using non-linear regression (rectangular binding hyperbola).

### Cell-Free Protein Expression

Cell-free protein expression was performed by a coupled *in vitro* transcription/translation system as previously described (Schwarz et al., 2007) using a bacterial S30 extract from *E. coli* BL21 (DE3), which already contains the T7-RNA polymerase.

The S30 extract was prepared manually. In short, an overnight culture of *E. coli* BL21 (DE3) in 100 mL TB-Medium was harvested. The cells were resuspended in 2 L 2x YTPG medium to obtain an OD_600_ of ∼0.1. The cells were allowed to grow at 37°C, 200 rpm to an OD_600_ of 0.6 and the expression of the T7-RNA Polymerase was induced by 1 mM IPTG (Thermo Fisher Scientific). At OD_600_ of 2.2–2.4 cells were harvested by centrifugation at 8,000 *g*, 4°C for 5 min. The pellets were washed three times with 10 mM Tris-acetate (pH 8.2, Carl Roth GmbH & Co. KG, Karlsruhe, Germany), 14 mM Mg(OAc)_2_ (Merck Millipore), 0.6 mM KCl (Grüssing GmbH Analytika, Filsum, Germany) and 6 mM β-mercaptoethanol (Sigma-Aldrich) and centrifuged at 8,000 *g*, 4°C for 5 min. Next, the pellet was resuspended in 1.2 mL 10 mM Tris-acetate (pH 8.2), 14 mM Mg(OAc)_2_, 0.6 mM KCl, 1 mM dithiothreitol (DTT, Thermo Fisher Scientific) and 1 mM Pefabloc SC (Sigma-Aldrich). The cells were disrupted by FastPrep-24^TM^ 5G (MP Biomedicals, Inc.) and the lysate centrifuged at 30,000 *g*, 4°C for 30 min. Centrifugation was repeated with the supernatant. The supernatant was collected, adjusted to a final concentration of 400 mM NaCl and incubated at 42°C for 45 min. Subsequently the turbid extract was dialyzed against 100 times expression buffer [10 mM Tris-acetate (pH 8.2), 14 mM Mg(OAc)_2_, 0.6 mM KOAc (Sigma-Aldrich) and 0.5 mM DTT] for 2 h at 4°C. Dialysis was repeated with fresh buffer over night. The extract was then centrifuged at 30,000 *g*, 4°C for 30 min, the supernatant was aliquoted and stored at -70°C. For each new S30 extract the optimal Mg(OAc)_2_ concentration has to be adjusted. CECF protein synthesis was performed as previously described (Schwarz et al., 2007) with volumes of 75 μL reaction mix (RM) and 1 mL feeding mix (FM) in the analytical scale and 1 mL RM and 17 mL FM in the preparative scale. The RM and FM consisted of the following components: 2% (w/v) polyethylene glycol 8000 (AppliChem GmbH, Darmstadt, Germany), 1 mM IPTG, 2 mM DTT, 150.8 mM KOAc, 10.1 mM Mg(OAc)_2_, 0.1 M HEPES, 1x Halt^TM^ Protease Inhibitor Cocktail (Thermo Fisher Scientific), 20 mM phosphoenolpyruvate (Bachem, Bubendorf, Switzerland), 0.05% (w/v) sodium azide, 0.1 mg/mL folinic acid, 1.2 μM ATP, 0.8 μM CTP, GTP and UTP (Sigma-Aldrich), 20 mM acetyl phosphate (Sigma-Aldrich), 0.5 mM of each amino acid (Sigma-Aldrich), supplemented with additionally 1 mM of arginine, cysteine, tryptophane, methionine, glutamic acid and aspartic acid. The FM consisted of additional 0.5 mM of each amino acid and 35% expression buffer. The RM was supplemented with 0.04 mg/mL pyruvate kinase, 0.5 mg/mL tRNA (Sigma-Aldrich), 0.3 U/μL RNaseOUT Recombinant Ribonuclease Inhibitor (Thermo Fisher Scientific), 0.015 μg/mL DNA and 35% (v/v) S30-extract.

For testing and optimization of the receptor expression, an analytical scale was used. Screening for the optimal expression temperature was accomplished by incubation at 27, 30, 33, 37, or 40°C for 16 h, respectively. To obtain a soluble receptor expression *n*-dodecyl β-D-maltoside (DDM, AppliChem GmbH) and Chaps (Carl Roth, Karlsruhe, Germany) were added to the RM and the feeding mix separately in final concentrations of 0.3% (w/v) and 0.6% (w/v). Elsewise, Brij-58 and Brij-35 (Sigma-Aldrich, Taufkirchen, Germany) were used in final concentrations of 0.01–0.5% (w/v). Furthermore, the influence of L-glutathione oxidized (GSSG), reduced (GSH) (Carl Roth) and cholesteryl hemisuccinate (CHS) (Sigma-Aldrich) addition and the pH value on receptor expression was tested.

To determine the influence of additives on receptor expression Western Blots were used comparing the receptor band intensity with and without additives. The amount of solubly expressed receptor was determined by comparing the receptor band intensity in the supernatant versus the intensity in the crude reaction. To measure receptor band intensities the software Gene Tools (Syngene, Cambridge, United Kingdom) was used.

Preparative scale expression was performed as previously described (Schwarz et al., 2007; Yang et al., 2018) scaling 1 mL RM and 17 mL feeding mix. Soluble membrane protein expression was achieved by addition of 0.1% (w/v) Brij-35 or Brij-58, 1 mM GSSG and 5 mM GSH. The expression was performed for 16 h at 37°C under constant shaking.

### Ligand Affinity Chromatography

After receptor expression the buffer was exchanged to a binding buffer [0.1 M Tris/HCl, pH 7.4, 5% glycerol and 0.1% (w/v) Brij-35/-58] and samples were purified by ligand affinity chromatography using biotin-(Ahx)_2_-NPY immobilized on Pierce Avidin Agarose beads (from Thermo Fisher Scientific) as previously described (Bosse et al., 2011; Yang et al., 2018). In short, the receptor was added to the immobilized biotin-(Ahx)_2_-NPY over night at 4°C repetitively by using a peristaltic pump P-1 (GE Healthcare). On the next day, the column was washed five times with one bed volume of binding buffer. Elution was performed by weakening the electrostatic ligand-receptor interactions using 60 mM CaCl_2_ in binding buffer or by replacing the immobilized ligand from the receptor binding pocket by addition of an excess (10^-5^ and 10^-6^ M) of soluble ligand in the binding buffer, each two times for 30 min at room temperature under constant agitation. Total protein concentrations before and after ligand affinity purification were determined by Bio-Rad Protein against a BSA standard.

### Functionality of Cell-Free Expressed Y_2_R

Functionality of cell-free expressed Y_2_R samples was verified by a homogenous binding assay based on fluorescence polarization as described previously (Yang et al., 2018). As fluorescent tracer, the Y_2_ selective NPY variant Ahx(5-24)-NPY (Cabrele and Beck-Sickinger, 2000) labeled with a 5(6)-TAMRA dye was used (5(6)-TAMRA-Ahx(5-24)NPY). In short, CaCl_2_ was removed from affinity purified Y_2_R_cysteine_deficient by dialysis and the concentration was determined by measuring A_280_ at a Tecan Spark plate reader (Tecan Group AG). Increasing concentrations of Y_2_R_cysteine_deficient in Brij-58 micelles in binding buffer were incubated with 5 nM 5(6)-TAMRA-Ahx(5-24)NPY for 60 min under gentle agitation in opaque 96-well plates. Fluorescence was measured in a Tecan Spark plate reader using linear polarized light. The excitation wavelength was adjusted to 510 nm (bandwidth 10 nm) and emission was measured at 590 nm (bandwidth 20 nm) perpendicular to the excitation light plane. Experiments were conducted at least twice independently in triplicates.

### Photo-Crosslinking Experiment Between Y_2_R and NPY Position 27

For photo-crosslinking Y_2_R_cysteine_deficient was purified by ligand affinity chromatography as previously described (Bosse et al., 2011). Elution was performed by replacing the immobilized biotin-(Ahx)_2_-NPY in the Y_2_R_cysteine_deficient binding pocket with the photoactivatable NPY variant. This was performed by incubating with 10^-5^ M Photo^27^-NPY in binding buffer for 30 min at room temperature while slightly shaking. The elution was repeated with 10^-6^ M Photo^27^-NPY. The eluted fractions were placed on ice and irradiated with UV light (UV lamp: AtlasFluotest forte, λ = 366 nm, 180 W) for 90 min 50 μl of photo-crosslinked Y_2_R_cysteine_deficient sample (∼20 μg) was digested with chymotrypsin (obtained from Promega, Mannheim, Germany) according to the manufacturer’s protocol. Crosslinked fragments were isolated by affinity purification using Monomeric Avidin Agarose beads (obtained from Thermo Fisher Scientific, Braunschweig, Germany) according to the manufacturer’s protocol.

Y_2_R_cysteine_deficient fragments after chymotrypsin digestion were calculated by the online tool PeptideMass (Wilkins et al., 1997). The selected enzyme was “Chymotrypsin (C-term to F/Y/W, not before P)”, for the cysteine treatment the option “Iodoacetamide” was chosen and the tool was allowed for up to five missed cleavages. The Photo^27^-NPY fragments were calculated manually by using the program ChemDraw (PerkinElmer, Waltham, MA, United States). Possible photo-crosslinked Y_2_R_cysteine_deficient – Photo^27^-NPY fragments were calculated by adding calculated Y_2_R_cysteine_deficient fragments to the Photo^27^-NPY fragments containing at least the amino acids NPY (22-27). Peptide fragments of photo-crosslinked Y_2_R_cysteine_deficient were analyzed by MALDI-ToF MS and MS/MS using an Ultraflex III MALDI-TOF/TOF mass spectrometer in reflector mode. In addition Y_2_R_cysteine_deficient alone and with Photo^27^-NPY and an excess of NPY in a molar ration of 4:1:8 were incubated at room temperature for 30 min and subsequently irradiated with UV-light for 90 min 50 μL of the samples were digested using chymotrypsin. The fragments were purified by monomeric Avidin Agarose beads and analyzed by MALDI-ToF MS to identify signals resulting from the receptor and unspecific photo-crosslinking. Analysis was performed manually for identification of photo-crosslinking fragments by MALDI-ToF MS. For identification of fragmentation by MS/MS, the software Biotools 2.2 (Bruker) was used, allowing crosslinking between Bpa at position 27 of NPY with one amino acid sequence of the identified receptor fragment at a time.

## Results

### Cell-Free Expression and Solubilization

**FIGURE 1:**
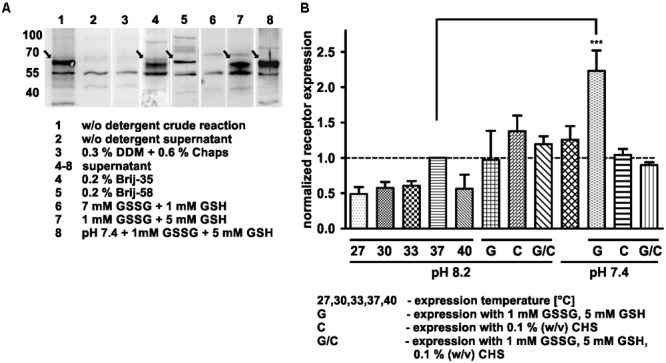
Optimization of cell-free Y_2_R_eGFP expression. **(A)** Western Blot of cell-free expressed hY_2_R_eGFP at different conditions (selected shown, spliced from different Western Blots). To monitor soluble receptor expression the crude reaction (1, 3) was centrifuged for 2 min at 13,000 rpm and the supernatant (2, 4–8) used for analysis. Without detergents (2), the receptor remains insoluble, while addition of DDM and Chaps (3) completely inhibits receptor expression. The addition of Brij-35 (4) or Brij-58 (5) led to high amounts of solubly expressed receptor. To promote disulfide bridge formation oxidized (GSSG) and reduced (GSH) glutathione were used. While a higher amount of GSSG inhibits soluble receptor expression (6), an increased amount of GSH promotes it (7). The effect was further increased by lowering the pH of expression buffer toward pH 7.4 (8). **(B)** Analysis of receptor expression based on fluorescence measurements and Western Blot analysis. All samples were normalized to receptor expression at 37°C, without additives, with expression buffer at pH 8.2. The receptor expression was tested at different temperatures (27, 30, 33, 37, and 40°C) without additives and at 37°C with 1 mM GSSG/5 mM GSH (G), 0.1% cholesteryl hemisuccinate (CHS, C) or with 1 mM GSSG, 5 mM GSH and 0.1% cholesteryl hemisuccinate (G/C) by using an expression buffer at pH 8.2 or 7.4. The expression in the presence of 1 mM GSSG and 5 mM GSH at pH 7.4 leads to a significantly increased receptor expression. Arrows indicate synthesized receptors. DDM, *n*-dodecyl β-D-maltoside; Chaps, 3-[(3-cholamidopropyl)dimethylammonio]-1-propansulfonat; Brij-35, polyoxyethylene(23)laurylether; Brij-58, polyoxyethylene(20)cetylether, *n* ≥ 2, ^∗∗∗^*P* < 0.005, one-way analysis of variance.

The aim of this study was to produce soluble Y_2_ receptor, either coupled to a C-terminal fluorescent protein or as a cysteine deficient variant, by an *E. coli* based CECF system. At first, Y_2_R_eGFP was expressed in an analytical scale in the presence of different detergents to ensure soluble expression. Receptor production after expression was monitored by Western Blot analysis (Figure 1A and Supplementary Figure S3) of the crude RM or its supernatant after 2 min centrifugation at 13,000 rpm. The amount of solubly expressed receptor was estimated by comparison of the receptor band intensity in the crude RM with the supernatant. In the absence of detergents the receptor is expressed, but remains insoluble. For a soluble expression, detergents were added during expression. The detergents 0.3% (w/v) DDM and 0.6% (w/v) Chaps, which have been used to refold the recombinantly expressed Y_2_ receptor (Schmidt et al., 2009), completely inhibited receptor expression in the CECF system. As an alternative the polyoxyethylene alkyl-ether Brij-35 and Brij-58, both with a critical micellar concentration below 0.1 mM, were added in final concentrations of 0.01–0.5% (w/v). These detergents have been used for soluble expression of a number of GPCRs by cell-free systems (Klammt et al., 2005; Schwarz et al., 2007; Corin et al., 2011a; Junge et al., 2011; Isaksson et al., 2012; Orban et al., 2015). Both detergents were able to solubilize Y_2_R_eGFP in high amounts, with Brij-35 leading to more than 90% of soluble receptor (Table 1), while the addition of Brij-58 led to ∼80% of soluble Y_2_R_eGFP. For Y_2_R_cysteine_deficient the solubilization by Brij-58 was more effective, resulting in approximately 80% of soluble receptor, compared to 55% of soluble receptor when using Brij-35 (Table 1). Since high concentrations of detergents can disturb structural investigations, the amount of detergents was reduced stepwise (data not shown). For Brij-35, a minimal concentration of 0.1% (w/v) is required for efficient receptor solubilization, while lower concentrations reduced the amount of solubly expressed Y_2_R_eGFP to ∼70%. By using Brij-58, a final concentration of 0.01% (w/v) is sufficient to solubilize 80% of expressed Y_2_R_eGFP.

**Table 1:** Soluble expression of Y_2_R_eGFP or Y_2_R_cysteine_deficient in presence of different detergents.

Detergent	CMC [mM]	Final concentration [%] (x CMC)	Amount of solubilized receptor [%]
DDM/Chaps	0.17/10	0.3/0.6 (33/1)	–^∗^	
Brij-35	0.09	0.1 (9)	Y_2_R_GFP	>90
			Y_2_R_cysteine_deficient	55
Brij-58	0.08	0.1 (12)	Y_2_R_GFP	80
			Y_2_R_cysteine_deficient	80

*Non-soluble protein was removed after expression by centrifugation. Soluble fractions were determined by Western Blot Analysis with subsequent measurement of the band intensity by GeneTools (Syngene) in the soluble fraction vs. the crude reaction mix after expression. ^∗^-protein expression was inhibited.*

However, to ensure a stable solubilization also in a larger scale, final concentrations of 0.2% (w/v) Brij-35 for the preparative scale expression of Y_2_R_eGFP or 0.1% (w/v) Brij-58 for the expression of Y_2_R_cysteine_deficient were used.

### Optimization of Receptor Expression

The cell-free expression system provides the advantage of easy adjustment to the requirements of the expressed protein. Thus, the Y_2_R_eGFP expression was optimized by changes in the expression temperature and pH value. The results have been normalized to the amount of receptor expressed at standard conditions (37°C, pH 8.2, w/o detergent) and are shown in Figure 1B and Table 2. A reduction of the expression temperature to 27°C, 30°C, 33°C or an increase to 40°C reduced the amount of solubly expressed Y_2_R_eGFP by about 50%. Adjusting the pH of the expression buffer toward the cytoplasmic pH of eukaryotic cells (Madshus, 1988) can promote the expression of eukaryotic proteins by cell-free systems. Therefore, a pH 7.4 was used during Y_2_R_eGFP expression, increasing the amount of expressed protein by about 25%.

**Table 2:** Optimization of cell-free Y_2_R_eGFP expression.

	Expression conditions	Receptor expression (fold over standard)^[a]^
	**Expression temperature**		
	27C	0.49	
	30°C	0.58	
	33°C	0.61	
	37°C	1.00	
	40°C	0.56	

**pH value**	**Additives**		

pH 8.2	GSSG, GSH	0.97	
	CHS	1.38	
	GSSG, GSH, CHS	1.19	
pH 7.4		1.26	
	GSSG, GSH	2.23	^∗∗∗^
	CHS	1.04	
	GSSG, GSH, CHS	0.90	

*Analysis of receptor expression based on fluorescence measurements and Western Blot. All samples were normalized to receptor expression at 37°C, without additives, with expression buffer at pH 8.2. To test for their influence on receptor expression 1 mM GSSG, 5 mM GSH or 0.1% (w/v) CHS were added to the reaction mix. ^[*a*]^Standard condition -37°C, buffer pH 8.2, 0.2% Brij-35 final, ^∗∗∗^*P* < 0.005 one-way analysis of variance.*

GPCRs contain several disulfide bridges that are important for correct folding and function. To promote formation of correct disulfide bridges oxidized (GSSG) and reduced (GSH) glutathione were added during soluble expression in presence of 0.2% Brij-35. When using 7 mM GSSG and 1 mM GSH the cell-free protein expression was strongly reduced, whereas the use of higher concentrations of GSH (5 mM) than GSSG (1 mM) promoted the receptor expression. Using the glutathione shuttle in combination with the reduced pH value significantly increased receptor expression up to twofold.

CHS is an acidic cholesterol ester, which assembles itself into bilayers and can be used in combination with detergents to enhance micelle stability and membrane protein folding (Tucker and Grisshammer, 1996). To elucidate whether CHS has a positive effect on the receptor expression and solubilization 0.1% (w/v) CHS was added to the RM in combination with pH 8.2 or pH 7.4, 0.2% Brij-35, as well as with and without addition of 1 mM GSSG and 5 mM GSH. However, none of these conditions led to a significant increase in receptor expression.

As for structural investigations larger amounts of receptor are needed, a preparative scale was used with the optimized conditions for soluble Y_2_R expression, determined in the analytical scale. An expression at pH 7.4 with 1 mM GSSG, 5 mM GSH and the respective detergent led to a stable and soluble receptor expression in the preparative scale and these conditions were used for further investigations.

### Purification and Functionality of Cell-Free Expressed Y_2_ Receptors

**FIGURE 2:**
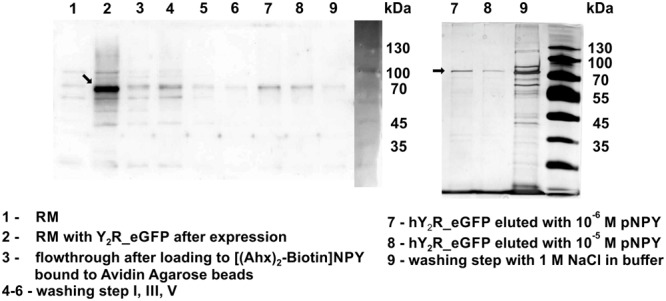
Western Blot **(left)** and silver staining **(right)** of cell-free expressed hY_2_R_eGFP before and after ligand affinity chromatography. The reaction mix (RM) was plotted before (1) and after (2) expression of Y_2_R_eGFP to determine a successful protein expression. Next, RM was added to biotin-(Ahx)_2_-NPY loaded columns (3) and unfolded/not correctly folded Y_2_R_eGFP was washed away by multiple washing steps (4–6). Next, correctly folded receptor was eluted specifically by adding an excess of NPY, Y_2_R_eGFP is represented by one band in lane 7, 8. After specific elution, residual Y_2_R_eGFP was washed from column by 1 M NaCl in buffer (9). Arrows indicate Y_2_R_eGFP.

For purification of the expressed receptors and to separate correctly folded from unfolded or misfolded Y_2_ receptors, ligand affinity chromatography was used as described by Bosse et al. (2011). For immobilization of NPY on Avidin Agarose beads NPY was biotinylated at its N terminus, which is not involved in ligand binding and signal transduction (Cabrele and Beck-Sickinger, 2000; Lindner et al., 2009) and can therefore be used for modifications. To ensure specific binding and elution an excess of soluble NPY was used to replace the biotin-NPY from the column. By comparing receptor band intensity in Western Blot analysis and silver stain (Figure 2) before and after purification by ligand affinity chromatography, an amount of about 25% correctly folded receptor was determined. The eluted fractions contain 0.78 mg Y_2_R_eGFP or 0.26 mg Y_2_R_cysteine_deficient, respectively, resulting from 1 mL of RM. This corresponds to a yield of 4.9% for Y_2_R_eGFP or a yield of 2.3% for Y_2_R_cysteine_deficient (Table 3).

**Table 3:** Purification of cell-free expressed Y_2_R_eGFP and Y_2_R_cysteine_deficient, resulting from 1 mL of reaction mix.

Step		Volume [mL]	Total protein^[a]^		Yield (%)
			[mg]	[nmol]	
Crude extract^[b]^	Y_2_R_eGFP	1	15.9		100
	Y_2_R_cysteine_deficient		11.5		100
Ligand affinity	Y_2_R_eGFP	3	0.78	10.11	4.9
chromatography	Y_2_R_cysteine_deficient		0.26	5.16	2.3

*^[*a*]^Total protein was determined by Bio-Rad Protein Assay using BSA as standard protein. ^[*b*]^Crude extract from 1 mL reaction mix, which correlates to one cell-free preparative scale expression.*

**FIGURE 3:**
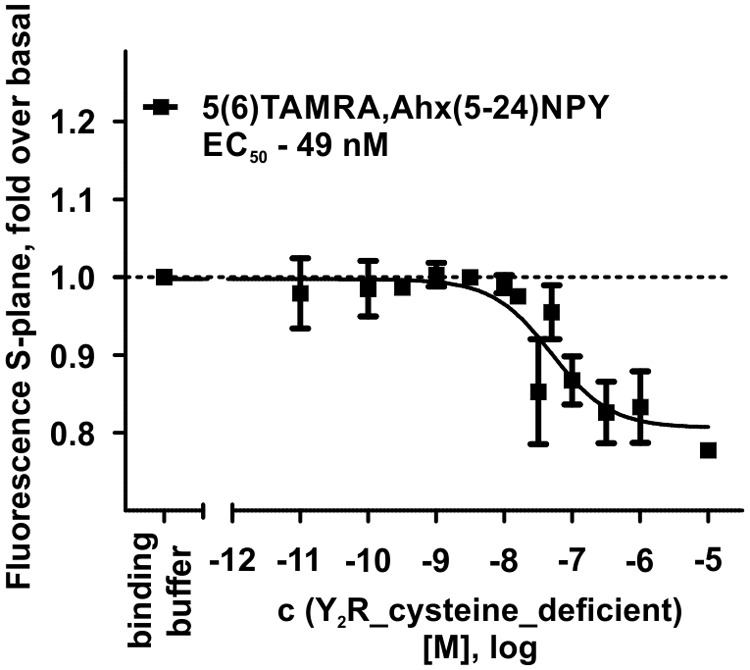
Fluorescence polarization assay of 5 nM 5(6)-TAMRA labeled, short NPY [5(6)-TAMRA-Ahx(5-24)NPY] with increasing amounts of cell-free expressed Y_2_R_cysteine_deficient in Brij-58 micelles. Data show a reduction in fluorescence S-plane upon 5(6)-TAMRA-Ahx(5-24)NPY binding after 60 min incubation at room temperature. The determined EC_50_ value was 49 nM. EC_50_ and pEC_50_ ± s.e.m. were determined using GraphPad Prism 5.0. All curves were baseline corrected to the S-Plane fluorescence without receptor (binding buffer) *n* ≥ 2.

**FIGURE 4:**
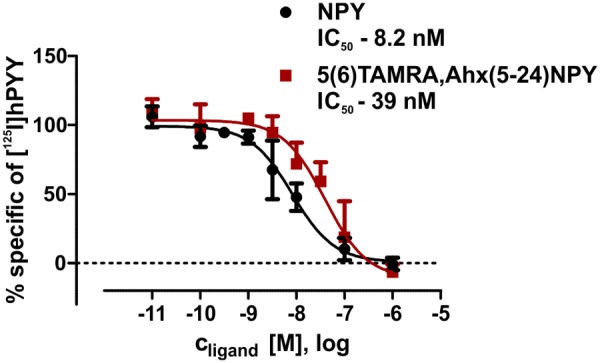
Summary of binding assays with membrane preparations of transiently transfected HEK293 cells. IC_50_ values were determined by displacement assay (60 pM [^125^I]pPYY) after incubation with peptidic ligands. All curves were normalized to the associated NPY curves. Values were calculated using GraphPad Prism 5.0 non-linear regression (curve fit) *n* ≥ 2.

The interaction between Y_2_R and NPY C terminus is strongly dependent on electrostatic interaction (Merten et al., 2007), therefore, the bivalent ion Ca^2+^ can be used to weaken the electrostatic NPY-receptor interactions, leading to an elution of the receptor from the column. An efficient elution of the receptor can be achieved with 60 mM calcium chloride in the binding buffer (Bosse et al., 2011). This has been used to purify Y_2_R_cysteine_deficient for the binding assay based on fluorescence polarization. Thus, affinity purified Y_2_R_cysteine_deficient was dialyzed to remove calcium chloride from the binding buffer and tested for its functionality by a homogenous binding assay based on fluorescence polarization. 5(6)-TAMRA labeled, Y_2_ selective (Kirby et al., 1993; DeCarr et al., 2007) NPY analog [5(6)-TAMRA-Ahx(5-24)NPY] was synthesized by solid phase peptide synthesis. Purity and identity were confirmed by RP-HPLC and MALDI-ToF MS (Table 4). Using an IP-one accumulation assay, 5(6)-TAMRA-Ahx(5-24)NPY was tested for its ability to activate Y_2_R_cysteine_deficient (Table 4 and Supplementary Figure S1). The ligand is able to fully activate the cysteine deficient Y_2_ receptor with a potency of 3.6 nM. To estimate binding of 5(6)-TAMRA-Ahx(5-24)NPY to the cell-free expressed Y_2_R_cysteine_deficient, a homogenous binding assay was used, which eliminates the necessity for separation of bound from unbound ligand. 5(6)-TAMRA-Ahx(5-24)NPY was incubated with cell-free receptor in Brij-58 micelles and changes in the emission of linearly polarized fluorescence was detected (perpendicular/S-plane). After ligand binding a reduction in the fluorescence in the S-plane was detected. The reduction showed a sigmoidal curve progression, displayed in Figure 3 and allowed the determination of the EC_50_ of 49 nM (pEC_50_ 7.3 ± 0.25) by logistic fitting, which constitutes the affinity constant (*K*_d_). To confirm the results and compare the binding constant with a mammalian expression, additional binding experiments were performed with membrane preparations of Y_2_R_cysteine_deficient in HEK293 cells and [^125^I]PYY. By a displacement assay we obtained an IC_50_ value of 39 nM (pIC_50_ 7.4 ± 0.19) for 5(6)-TAMRA-Ahx(5-24)NPY, Figure 4, which results in an *K*_i_ value of 11.2 nM for 5(6)-TAMRA-Ahx(5-24)NPY (*K*_d_ value [^125^I]PYY 24.1 pM ± 8.4, data not shown). The EC_50_ value of the cell-free expressed Y_2_R_cysteine_deficient is only about four times higher than the *K*_i_ value determined by the radioactive binding assay. Consequently, cell-free expressed Y_2_R_cysteine_deficient is functional and can be used for photo-crosslinking experiments.

**Table 4:** Analytical characterization of NPY analogs for ligand affinity chromatography, crosslinking experiments and binding assays.

Peptide	Chemical formula	*M*_calc._ [Da]	MALDI-ToF MS [M + H]^+^ [Da]	Purity [%]^[a]^	EC_50_ [nM] (pEC_50_ ± s.e.m.)^[b]^	*E*_max_± s.e.m. [%]^[c]^
NPY	C_190_H_287_N_55_O_57_	4251.1	4252.0	>95	0.1 (9.79 ± 0.15)	92 ± 4
Biotin-(Ahx)_2_-NPY	C_212_H_323_N_59_O_61_S	4703.4	4704.3	>95	1,2 (8.91 ± 0.25)	98 ± 8
5(6)-TAMRA,Ahx(5-24)NPY	C127H_182_N_34_O_28_	2631.4	2632.4	>95	3.4 (8.42 ± 0.21)	95 ± 8
[K^22^[(Ahx)_2_-biotin]Bpa^27^]NPY	C_222_H_334_N_60_O_60_S	4832.5	4833.4	>95	4.0 (8.40 ± 0.25)	102 ± 12

*The influence on Y_2_R_cysteine_deficient signal transduction was determined by an IP-one assay. ^[*a*]^The purity of all peptides was determined by analytical reverse phase HPLC at 220 nm by two different columns. ^[*b,c*]^EC_*50*_ and pEC_*50*_ ± s.e.m. (standard error of the mean) and E_*max*_ ± s.e.m. were determined using GraphPad Prism 5.0. All curves were normalized to the top and bottom values of the Y_*2*_R/NPY curve. Non-linear regression (curve fit) was performed for normalized response in all assays n ≥ 2*.

### Photo-Crosslinking Between Y_2_R_Cysteine_Deficient and Position 27 of NPY

**FIGURE 5:**
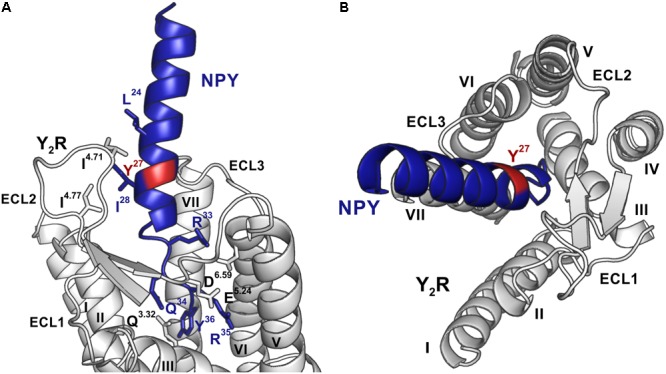
Model of NPY bound to Y_2_R, modified from Kaiser et al. (2015). Position 27, which was used for introduction of the photo-crosslinker, is highlighted in red, NPY is shown in blue and Y_2_R in gray. **(A)** The C-terminus of NPY unwinds upon binding to the TM helix bundle, while I^24^ and I^28^ of NPY form hydrophobic contact toward the ECL2 of Y_2_R. The side-chains of residues involved in binding are shown in the respective colors and are labeled. **(B)** NPY has a steep binding mode, as viewed from the top, with the C-terminus of NPY binding deep inside the TM helix bundle. ECL, extracellular loop; TM, transmembrane.

**FIGURE 6:**
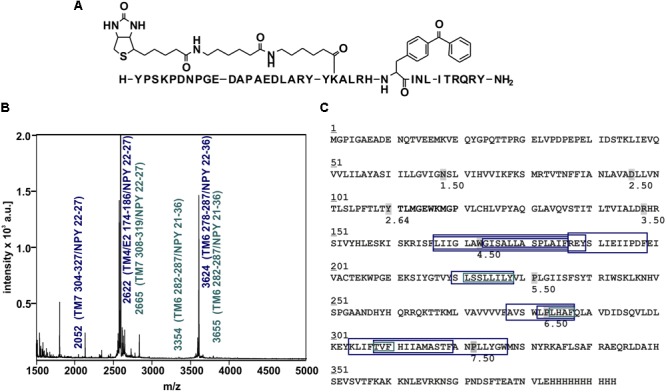
Mass spectra of photo-crosslinked Y_2_R_cysteine_deficient with [K^22^[(Ahx)_2_-biotin]Bpa^27^]NPY and respective regions of NPY at Y_2_R_cysteine_deficient. **(A)** Structure of [K^22^[(Ahx)_2_-biotin]Bpa^27^]NPY. **(B)** Exemplary MALDI-ToF mass spectrum of photo-crosslinked samples enzymatically digested with chymotrypsin. Potential Y_2_R_cysteine_deficient fragments photo-crosslinked with [K^22^[(Ahx)_2_-biotin]Bpa^27^]NPY are labeled in blue, fragments which were analyzed by tandem MS are labeled in cyan. TM, transmembrane helix; E, ECL; NPY, [K^22^[(Ahx)_2_-biotin]Bpa^27^]NPY. **(C)** Amino acid sequence of Y_2_R_cysteine_deficient with C-terminal deca-histidine-tag. The detected regions of Y_2_R_cysteine_deficient after crosslinking with [K^22^[(Ahx)_2_-biotin]Bpa^27^]NPY are marked with boxes (blue). Regions analyzed by MS/MS are emphasized by cyan boxes. The different sizes of the boxes represent different detected fragments. All experiments were repeated at least two times, independently.

Photo-crosslinking can be used to investigate binding motifs between ligands and their respective targets. As shown by double cycle mutagenesis (Kaiser et al., 2015) L^24^ and I^28^ of the ligand bind to the ECL2 of Y_2_R by hydrophobic contacts, furthermore the C-terminal binding of NPY to Y_2_R has been intensively investigated. Based on these data a model of NPY docked into Y_2_R was generated by Kaiser et al. (2015). Figure 5 shows the homology model, which was adapted from this reference. For Y^27^ in the α-helical part of NPY no direct interactions has been suggested by the model. Therefore, NPY analogs carrying the highly reactive Bpa (Photo^27^-NPY) were synthesized by solid phase peptide synthesis and tested in cell-based *in vitro* assays (Table 4). To ensure photo-crosslinking only with functional receptors, cell-free expressed Y_2_R_cysteine_deficient in Brij-58 micelles was loaded on biotin-(Ahx)_2_-NPY columns, specifically eluted with 1 μM or 10 μM Photo^27^-NPY, respectively, and photo-crosslinked. The crosslinked complex was digested using chymotrypsin (Supplementary Figure S2) and resulting fragments were analyzed by MALDI-ToF MS (Figure 6 and Table 5). The signals found in the mass spectra, subtracted by signals of the digested receptor alone and bound to native NPY, were compared to the calculated masses of the expected photo-crosslinked fragments.

**Table 5:** Signals of photo-crosslinked Y_2_R_cysteine_deficient with [K^22^[(Ahx)_2_-biotin]Bpa^27^]NPY identified by MALDI-ToF mass spectrometry that correlate with the calculated masses of photo-crosslinked fragments of Y_2_R_cysteine_deficient with [K^22^[(Ahx)_2_-biotin]Bpa^27^]NPY and [K^22^[(Ahx)_2_-biotin]Bpa^27^]NPY with itself and are found in at least two independent experiments.

MALDI-ToF MS[m/z]^[a]^	Number in Y_2_R_ cysteine_deficient	Position in Y_2_R_ cysteine_deficient	Position NPY	*M*_calc._ [Da]^[b]^	[*M*_calc_ + H]^+^ [Da]^[c]^	[*M*_calc_ + Na]^+^ [Da]^[d]^	[*M*_calc_ + K]^+^ [Da]^[e]^
2051.9	304–327	7.32–7.55	22–27	4080.2	4081.2	4103.2	4119.2
2621.6	174–186	4.51–4.63	22–27	2599.5	2600.5	2622.5	2638.4
2665.5	308–319	7.36–7.48	22–27	2664.4	2665.4	2687.4	2703.4
			22–36 + 21–21	2664.5	2665.5	2687.5	2703.5
			21–27 + 28–36	2664.5	2665.5	2687.5	2703.4
2845.2	187–198	4.64–4.75	22–27	2821.5	2822.5	2844.5	2860.5
3345.0	282–287	6.49–6.54	21–36	3343.9	3344.9	3366.9	3382.8
3624.2	278–287	6.45–6.54	22–36	3623.0	3624.0	3646.0	3662.0
3655.1	220–228	5.39–5.47	21–36	3654.1	3655.1	3677.1	3693.0
3836.9	88–110	2.43–2.64	22–27	3836.1	3837.1	3859.1	3875.1
	167–189	4.44–4.66	22–27	3814.2	3815.2	3837.2	3853.1
4102.6	304–327	7.32–7.55	22–27	4080.2	4081.2	4103.2	4119.2
4524.8	167–186	5.35–5.62	22–36	4522.6	4523.7	4545.6	4561.6

*^[*a*]^Determined signals by MALDI-ToF MS. ^[*b*]^Correlated position in [K^*22*^[(Ahx)_*2*_-biotin]Bpa^*27*^]NPY (designated NPY). ^[*c*-*e*]^Selected calculated masses in Dalton of possible photo-crosslinked fragments of Y_*2*_R_cysteine_deficient with [K^*22*^[(Ahx)_*2*_-biotin]Bpa^*27*^]NPY or [K^*22*^[(Ahx)_*2*_-biotin]Bpa^*27*^]NPY with itself. The fragments are selected based on the correlation with the detected signals. For clarity, further calculated masses of possible photo-crosslinked fragments are not shown*.

**FIGURE 7:**
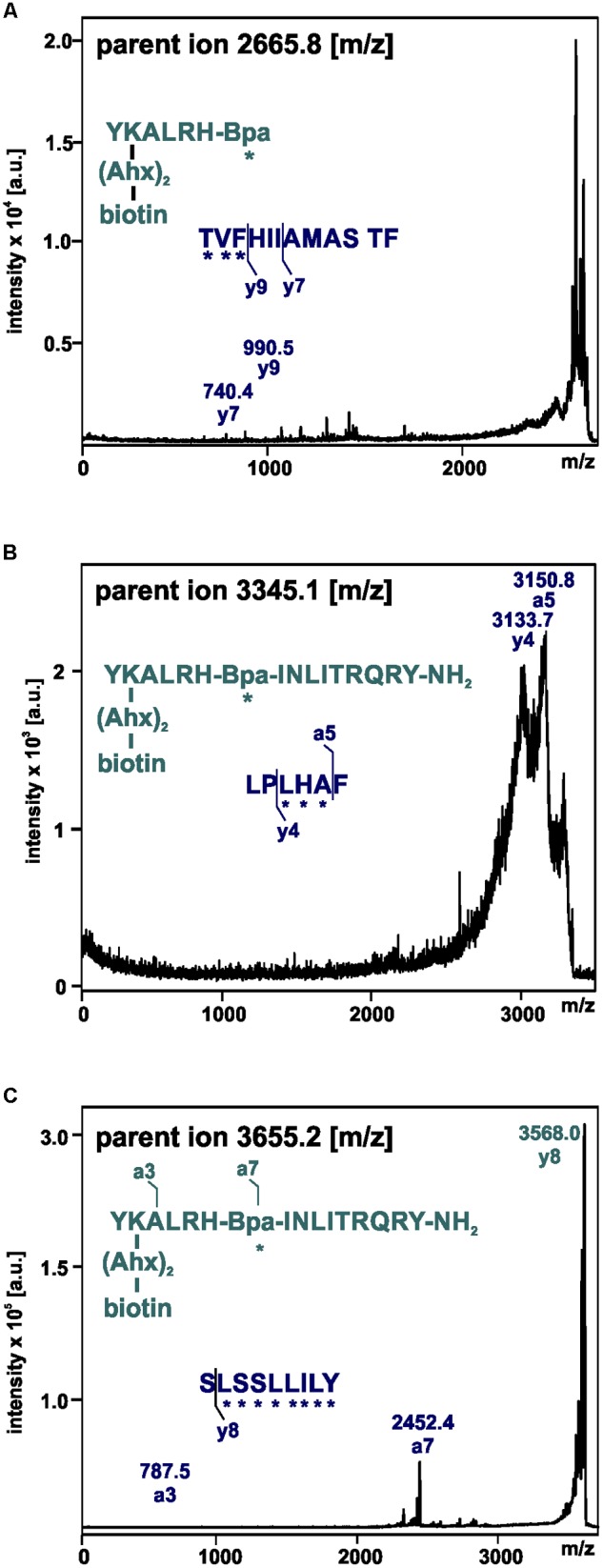
Mass spectra of MS/MS analysis of the signals m/z 2665.8 **(A)**, 3345.1 **(B)**, and 3655.2 **(C)** resulting from photo-crosslinked Y_2_R_cysteine_deficient with [K^22^[(Ahx)_2_-biotin]Bpa^27^]NPY. Amino acid sequences of the identified regions in Y_2_R_cysteine_deficient (blue) and [K^22^[(Ahx)_2_-biotin]Bpa^27^]NPY (cyan) are depicted with the fragmentation sites resulting from collision induced dissociation (y) or electron detachment dissociation (a). Mass spectra were analyzed using Biotools 2.2. ^∗^ potential site of photo-crosslinking.

**FIGURE 8:**
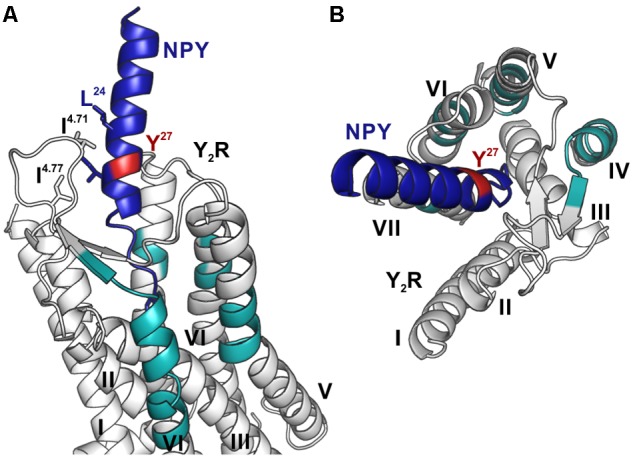
Model of NPY bound to Y_2_R. Fragments, which were identified by photo-crosslinking studies in combination with MALDI-ToF MS and tandem MS/MS, between position 27 (red) of NPY (blue) and Y_2_R_cysteine_deficient (gray) are labeled in cyan. The model is based on the model by Kaiser et al. (2015). **(A)** Position 27 of NPY binds toward the top of TM 5, 6, 7 as well as to the top of TM 4 and ECL2, reflecting the flexibility of this position, which has no fixed interaction partner. **(B)** Top view of NPY binding to Y_2_R.

These fragments were assigned to three different regions in the Y_2_R_cysteine_deficient, the outer parts of transmembrane helices (TM) 2 (I88–Y110), 5 (S220–Y228), 6 (A278–F287), 7 (K304–W327) and parts of TM4 and ECL2 (L167–Y189, R187–F198). One mass (m/z 3836.9) could be assigned to either I88–Y110 or to L167–Y189 of the receptor. As no additional signals support an interaction between Y^27^ of the ligand and I88–Y110 of the receptor an interaction with L167–Y189 is favored. To narrow down the interaction site for position 27 we performed tandem MS/MS analysis of the signals 2665.5, 3345.0, and 3655.1 (m/z). The results are shown in Figure 6, 7 and Supplementary Figure S4. We demonstrate that position 27 of NPY crosslinks to the amino acids T304, V305, and F307 in TM 7 and therefore exclude that the signal at m/z 2665.5 results from two Photo^27^-NPY crosslinked to each other. By tandem MS/MS of the signal 3345.0, we identified the amino acids L284, H285, or A286 in TM 6 as being photo-crosslinked to position 27 of NPY. By fragmentation of m/z 3655.1, we can rule out S220 as site for photo-crosslinking, leading to the fragment L221–Y228 in TM5 as potential crosslinking site. Unfortunately, we were not able to perform MS/MS analysis of the signals, which have been assigned to the upper part of TM4 and ECL2 of the receptor. The core regions of the detected signals (G174–Y189, L222–Y228, L284–A286, and T304–F307) were labeled in the model of Y_2_R bound to NPY(13–36) (Figure 8).

These results indicate that there is no fixed interaction area for Y^27^ of the ligand, but Y^27^ remains flexible after binding to Y_2_R_cysteine_deficient and photo-crosslinking occurs with regions of the receptor, which come into proximity to the ligand by fluctuations in the ECL2 and in the TM region. Furthermore, Y^27^ could bind to the transmembrane helices of Y_2_R_cysteine_deficient subsequent to ligand binding in the suggested two-step binding mode of NPY to Y_2_R.

## Discussion

Mass spectrometry based methods have gained more and more importance in the field of structural biology over the last years, since the instruments became more sensitive and new software for analysis is available. Crosslinking experiments are frequently used to search for unknown binding partners, to study conformational changes in proteins (Sinz, 2018) and also to map and identify binding sites *in vivo* (Seidel and Coin, 2018), as well as *in vitro* (Dehling et al., 2016; Kashiwagi et al., 2016). Thus, combined crosslinking and MS offer a complementary method to the classical structural methods like NMR spectroscopy or X-ray crystallography. In this study, we present a set-up for the *in vitro* production of homogenous, soluble and folded rhodopsin-like GPCRs by CF synthesis, exemplarily performed for Y_2_R. These receptors have been used for photo-crosslinking studies in combination with MS to identify binding sites.

The challenges in GPCR expression lie within their hydrophobic and flexible nature. Different protocols have been established for the recombinant production of GPCRs from prokaryotic expression hosts (Roy et al., 2008; Schmidt et al., 2009; Tapaneeyakorn et al., 2010; Shimamura et al., 2011) and these often contain several resolubilization, refolding, purification and stabilization steps. Therefore, this procedure is time consuming and the initial optimization process to produce large amounts of correctly folded GPCRs is labor intensive.

An alternative approach for the fast production of GPCRs are CF expression systems, which are frequently used for the expression of biomolecules otherwise hard to express, like toxic proteins (Orth et al., 2011; Villate et al., 2012), vaccines and small bioactive peptides (Lee et al., 2010; Tsuboi et al., 2010) and membrane proteins.

The expression of the Y_2_R by the CECF system was optimized with respect to expression temperature, buffer pH, detergents and additives as shown in Figure 1 and Tables 1, 2. This optimization was performed in an analytical scale. For co-translational solubilization of GPCRs expressed by an *E. coli* based CF system polyoxyethylene ethers are widely used, which are rather mild detergents. With these derivatives different GPCRs were successfully solubilized, including the human β2 adrenergic receptor, the human muscarinic acetylcholine receptor M2, the rat neurotensin receptor (Ishihara et al., 2005), the human endothelin B receptor and neuropeptide Y_4_ receptor, the human (Klammt et al., 2007), and rat vasopressin type 2 receptor (Klammt et al., 2005), the human trace amine-associated receptor 5 (Wang et al., 2013), the human endothelin A receptor (Junge et al., 2011), as well as different olfactory receptors (Corin et al., 2011b). We demonstrate that the use of either 0.2% (w/v) Brij-35 for Y_2_R_eGFP, or 0.1% (w/v) Brij-58 for the cysteine deficient Y_2_R, yields solubilization of about 80% receptor during expression. Protein expression is strongly dependent on the expression temperature, which has to be optimized for different proteins. We tested five different expression temperatures, as shown in Table 2. Compared to the initial expression temperature of 37°C, receptor expression was strongly reduced when lowering or increasing the temperature. As a stable redox potential is crucial for prolonged protein expression, a glutathione shuttle was added during expression, which has been shown to be beneficial for the expression of disulfide-containing proteins (Bundy and Swartz, 2011; Michel and Wüthrich, 2012). We further demonstrate that the addition of 1 mM GSSG and 5 mM GSH at a buffer pH of 7.4 leads to a twofold increased receptor expression compared to standard conditions. These optimized expression conditions were applied to the preparative scale expression for functional and structural investigations.

Ligand binding abilities of the expressed receptors have been monitored by ligand affinity chromatography, which was also used for purification and separation of correctly folded and unfolded or misfolded receptors. The elution was achieved by addition of an excess of soluble NPY, whereby the receptors are eluted specifically by competition of the immobilized ligand. Based on ligand affinity chromatography an amount of about 25% of correctly folded receptors was estimated (Figure 2), which corresponds to 0.78 mg Y_2_R_eGFP and 0.26 mg Y_2_R_cysteine_deficient per 1 mL of RM. This is comparable to the amount of folded receptors achieved by recombinant expression with subsequent *in vitro* folding (Bosse et al., 2011). With costs for the production of 1 mg correctly folded receptor varying from 50 € for Y_2_R_eGFP to 190 € for Y_2_R_cysteine_deficient this method is more expensive than the expression in *E. coli*. Nevertheless, cell-free expression techniques are very effective when considering labeling with, for example, isotopic labeled amino acids, which can be added in quite low amounts. Per preparative scale expression (1 mL RM) 34 nmol for R, C, W, M, D, and E or 18 nmol of the other amino acids are needed. For isotopic labeling a further optimization, especially for Y_2_R_cysteine_deficient, should be considered to reduce the costs for the production of 1 mg folded receptor.

To confirm the activity of affinity purified Y_2_R_cysteine_deficient, we aimed to perform binding assays. One problem to consider, when dealing with proteins solubilized in small detergent micelles, is the application of heterologous assays, since the micelles are too small for most filtration techniques. An alternative approach is the immobilization of the protein on functionalized beads, which, however, leads to high unspecific binding, especially when dealing with sticky peptidic ligands like NPY. Therefore, a homogenous binding assay based on fluorescence polarization was used, as already performed for the CF expressed Y_1_R (Yang et al., 2018). For the Y_2_R_cysteine_deficient we used a 5(6)-TAMRA-labeled, Y_2_R selective NPY analog. By receptor binding the ligand is restricted in its movement, which can be observed by changes in the fluorescence S-plane. Using different concentrations of Y_2_R_cysteine_deficient in Brij-58 micelles, an EC_50_ value of 49 nM has been determined (Figure 3). By radioactive binding experiments the *K*_i_ value of 5(6)-TAMRA-Ahx(5-24)NPY was determined with 11.2 nM (Figure 4). The EC_50_ value determined by fluorescence polarization assay (which equals the *K*_d_ value of this experiment) is about four times higher than the determined *K*_i_ value. This very mild reduction of affinity likely originates from the artificial surrounding the Y_2_R_cysteine_deficient is folded into. Nonetheless, we were able to prove that the CF expressed, cysteine deficient Y_2_R in Brij-58 micelles is able to bind the ligand and that the binding is saturable. Therefore, we assume a correct folding of the receptor.

These findings demonstrate that the soluble CF expression is an efficient alternative to existing expression and refolding protocols, since the screening for suitable detergents can be performed in parallel in small scales. After identification of a suitable detergent the expression and solubilization can be achieved within 1 day. In contrast, expression of GPCRs in *E. coli* with subsequent, isolation and refolding protocols include several purification and solubilization steps. Most strategies use an initial solubilization in strong, denaturating detergents like sodium dodecyl sulfate, following an exchange to mild detergents or amphipols (Schmidt et al., 2009, 2017; Banères et al., 2011). For CF based methods optimization can be done straight forward for protein stabilizing agents, as well as for redox conditions as reviewed (Bernhard and Tozawa, 2013; Hoffmann et al., 2018).

We performed photo-crosslinking studies between the affinity purified Y_2_R_cysteine_deficient and Photo^27^-NPY. The benzophenone moiety used in Photo^27^-NPY can be activated at biocompatible wavelengths of 360 nm, which lowers the risk of damaging biomolecules (Dormán and Prestwich, 1994; Weber and Beck-Sickinger, 1997; Smith and Collins, 2015). Benzophenone was incorporated into the peptidic ligand NPY at position 27 as Bpa building block during the SPPS. This replacement is tolerated by the Y_2_ receptor, leading to only a minor loss of affinity (Beck-Sickinger et al., 1994; Cabrele and Beck-Sickinger, 2000). In addition to the crosslinking moiety, a biotin label was introduced at position 22 of NPY by a lysine side chain and a spacer made up from two Ahx residues. This allows separation of photo-crosslinked Y_2_R_cysteine_deficient fragments from non-crosslinked parts of the receptor by affinity purification exploiting the high affinity binding of biotin to avidin and streptavidin (Wilchek and Bayer, 1988; Dundas et al., 2013). This reduces the number of signals detected in the mass spectra. Furthermore, Brij-58 micelles can be washed from the photo-crosslinked fragments, since it disturbs the measurement by MS, leading to high, repetitive signals in the range of 500–1,500 Da.

To ensure specific photo-crosslinking, the Y_2_R_cysteine_deficient was loaded on immobilized biotin-(Ahx)_2_-NPY and unbound or weakly bound receptor was washed from the column. The receptor was eluted by displacing the immobilized biotin-(Ahx)_2_-NPY with Photo^27^-NPY. Subsequently the samples were photo-crosslinked, digested and affinity purified. Identification of residual Y_2_R_cysteine_deficient fragments after affinity purification was achieved by digesting the receptor without ligand with subsequent affinity purification and MS measurement. To exclude false identification of interaction sites between Y_2_R_cysteine_deficient and Photo^27^-NPY by unspecific photo-crosslinking, UV excitation was performed in presence of an excess of NPY, which should block the specific binding site in the receptor. Remaining Photo^27^-NPY can only unspecifically photo-crosslink with the receptor under those conditions. These signals were identified by MS and have been excluded from the analysis of the binding site.

Based on the signals in the mass spectra after photo-crosslinking, four regions of Y_2_R have been identified for making variable contacts with position 27 in NPY (Figure 6 and Table 5). In addition, we performed MS/MS tandem analysis of three of the identified regions to narrow the number of potential interaction points (Figure 7 and Supplementary Figure S4). This leads to the following sites for photo-crosslinking of position 27 of NPY: the outer parts of TM5 (L221–Y228), 6 (L284–F287), 7 (T304–F307) and parts of TM4 and ECL2 (core region G174–Y189). These findings are in agreement with the suggested binding mode of NPY on Y_2_R, which has been widely investigated by NMR spectroscopy, mutagenesis studies, as well as molecular modeling, leading to a model of Y_2_R bound to NPY(13–36) (Figure 5) (Kaiser et al., 2015). On Y_2_R, NPY adopts a steep binding mode, with the last C-terminal amino acids binding deep inside the TM helix bundle. This C-terminal part of NPY [NPY(25–36)] is crucial for the binding to Y_2_R and unwinds upon binding to the receptor. The N-terminal part of NPY can be truncated without affecting the affinity, which is different from the other receptors of the NPY family (Kirby et al., 1993; Beck-Sickinger and Jung, 1995; Cabrele and Beck-Sickinger, 2000; Cabrele et al., 2001; DeCarr et al., 2007). Moreover, the Y_2_R also tolerates an exchange of a part of the amphipathic α-helix ([Ahx(5-24)]NPY) (Kirby et al., 1993). Two positions in the amphipathic α-helix of NPY (namely I^24^ and I^28^) make hydrophobic contacts to the flexible ECL2 of Y_2_R (Kaiser et al., 2015). These contacts seem to guide the unwinding of the last five C-terminal residues to contact the deep binding pocket.

Y^27^ of NPY has no fixed interaction region, but is important for the structural integrity of NPY, since bulky amino acids are well tolerated at this position (Beck-Sickinger et al., 1994; Cabrele and Beck-Sickinger, 2000). It has been stated, that the position of NPY in the binding pocket of Y_2_R is not static (Kaiser et al., 2015) and may follow the movement of the ECL2, which is highly flexible. Based on our findings we propose, that the flexibility of NPY brings position 27 in proximity to the TM’s 4–7, allowing crosslinking to occur on variable parts of the receptor.

It is important to note, that by crosslinking approaches always an ensemble of conformations is identified (Sinz, 2018), as also seen during photo-crosslinking between Y_1_R and [Bpa^1^,K^4^[(Ahx)_2_-biotin]]NPY (Yang et al., 2018). In this study fragments found in the mass spectra, have been assigned to two different regions in Y_1_R: The ECL2 – which is the binding partner for the NPY N terminus, as suggested by modeling and mutagenesis studies, and the N terminus of Y_1_R, which is not necessary for receptor activation but significantly reduces NPY binding affinity when being removed (Lindner et al., 2009). For NPY and PYY a two-step binding mechanism has been proposed. In the first step monomeric NPY associates with the micellar/lipid surrounding, which forms hydrophobic contacts with the α-helix (Bader et al., 2001; Lerch et al., 2002; Thomas et al., 2005, 2009). The second step involves the entrance of NPY through the helices into the binding pocket (Bettio et al., 2002; Lerch et al., 2004; Thomas et al., 2005). The N terminus of Y_1_R seems to be involved in this binding mechanism of NPY and important for guidance of the ligand into the binding pocket. This entrance is also “captured” by the photo-crosslinking approach. The regions identified by photo-crosslinking between Photo^27^-NPY and Y_2_R also reflect different conformations of NPY during binding, as well as in the final binding mode. Y^27^, without fixed interaction partner, is able to crosslink with all regions, which come in proximity during the binding mechanism. This binding involves an additional step, the unwinding of the C-terminal NPY residues and binding in the deep binding pocket. During this mechanism, Y^27^ is also repositioned relative to Y_2_R, which is reflected by the different regions identified by MS after photo-crosslinking.

Overall our findings are in agreement with the proposed binding mode of Y_2_R to NPY(13–36) and demonstrate the robustness of the combined approach of CF synthesis and photo-crosslinking with subsequent MS. Y^27^ has no fixed interaction partner when bound to Y_2_R, but is important for the structural integrity of the peptide. Thus, our approach confirmed the proposed binding mode and no single interaction site was found, but an ensemble of different sites, which come into proximity during binding and activation of the receptor. This shows that our approach leads to no false-positive interaction sites, but, when carried out precisely, also shows the flexibility of some regions.

In this study, cell-free expressed Y_2_R was used for interaction studies by photo-crosslinking in combination with MS. We were able to successfully produce soluble, folded and functional Y_2_R by an *E. coli* based CECF system. The expression was optimized toward expression amount and receptor solubilization. By affinity purification 0.78 mg of pure, folded Y_2_R_eGFP or 0.26 mg Y_2_R_cysteine_deficient were isolated. By homogenous binding assay, functionality of Y_2_R_cysteine_deficient was confirmed and photo-crosslinking studies between Photo^27^-NPY and CF expressed receptor were performed. We demonstrate that Y^27^NPY remains flexible during binding to Y_2_R, since photo-crosslinking has been observed with different regions in TM5–TM7, as well as TM4 and ECL2. This is in agreement with the proposed binding model of NPY(13–36) with Y_2_R.

The cell-free expression is a powerful tool, enabling a fast and straight forward labeling of the expressed proteins site-specific with unnatural or isotopically labeled amino acids (Staunton et al., 2006; Bundy and Swartz, 2010; Su et al., 2011; Hong et al., 2014). Incorporation of photoactive amino acids, like photo-leucine or photo-methionine (Suchanek et al., 2005) by the *E. coli* translation machinery or the site-specific incorporation of unnatural photo-activatable amino acids like Bpa can be easily addressed. The cysteine deficient Y_2_ receptor variant, expressed by cell-free systems, offers the possibility of labeling by selective re-introduction of cysteines. This enables to perform labeling for photo-crosslinking experiments also from the receptor site by cell-free expression, giving further insights into interaction sites. Furthermore, site-specific introduction of amino acids into the protein allows for the introduction of other functionalities for structural investigations. This includes the possibility to introduce isotopically labeled amino acids for NMR spectroscopy or spin label for electron paramagnetic resonance spectroscopy in GPCRs in a fast and efficient way by cell-free expression.

## Data Availability

All datasets generated for this study are included in the manuscript and/or the Supplementary Files.

## Author Contributions

LK and AK performed the experiments. LK wrote the manuscript. JS and AB-S supervised the experiments and corrected the manuscript. AB-S conceived the experiments. All authors analyzed the data.

## Conflict of Interest Statement

The authors declare that the research was conducted in the absence of any commercial or financial relationships that could be construed as a potential conflict of interest.

## Abbreviations

5(6)-TAMRA: 5(6)-carboxytetramethylrhodamine
Ahx: 6-amino-hexanoic acid
ATP: adenosine-5′-triphosphate
Bpa: *p*-benzoyl-phenylalanine
Brij-35: polyoxyethylene(23)laurylether
Brij-58: polyoxyethylene(20)cetylether
BSA: bovine serum albumin
CECF: continuous exchange cell-free
Chaps: 3-[(3-cholamidopropyl)dimethylammonio]-1-propansulfonat
CHS: cholesteryl hemisuccinate
CTP: cytidine-5′-triphosphate
Da: dalton
DDM: *n*-dodecyl β-D-maltoside
GSH: L-glutathione reduced
GSSG: L-glutathione oxidized
GTP: guanosine-5′-triphosphate
HEPES: 2-[4-(2-hydroxyethyl)piperazin-1-yl]ethanesulfonic acid
IPTG: isopropyl β-D-1-thiogalactopyranoside
MALDI-ToF: matrix-assisted laser desorption ionization time of flight
MS: mass spectrometry
NMR: nuclear magnetic resonance
NPY: neuropeptide Y
PEG: polyethylene glycol
Photo^27^-NPY: [K^22^[(Ahx)_2_-biotin]Bpa^27^]NPY
PYY: peptide YY
RM: reaction mix
TM: transmembrane helix
tRNA: transfer ribonucleic acid
UTP: uridine-5′-triphosphate
Y_x_R: neuropeptide Y receptor type x
