# Supplementary material for: Cell-Free Expression and Photo-Crosslinking of the Human Neuropeptide Y2 Receptor
Source: Front Pharmacol. 2019 Mar 1;10:176. doi: 10.3389/fphar.2019.00176 (PMC6405639; doi:10.3389/fphar.2019.00176)
Supplement: Supplementary file 1 [file Data_Sheet_1.docx]

Supplementary Material

Cell-Free expression and Photo-Crosslinking of the Human Neuropeptide Y_2_ Receptor

Lisa Maria Kögler^1^, Jan Stichel^1^, Anette Kaiser^1^, Annette G. Beck-Sickinger^1^

^1^Institute of Biochemistry, Faculty of Life Sciences, Leipzig University, Brüderstr. 34, D 04103 Leipzig, Germany

*** Correspondence:**Annette G. Beck-Sickinger
abeck-sickinger@uni-leipzig.de


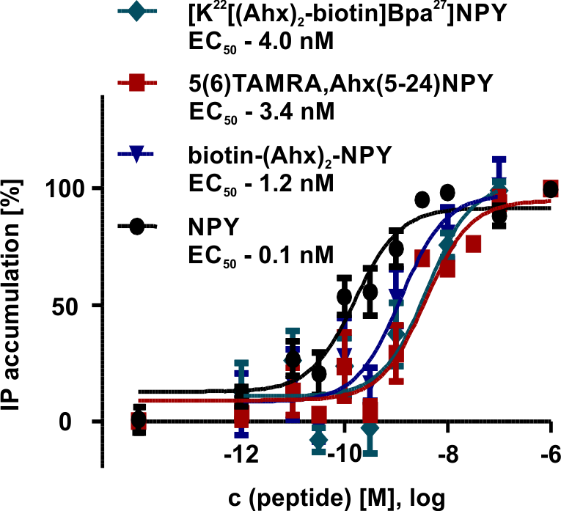


**Supplementary Figure S1.** Summary of IP accumulation assays with increasing concentrations of NPY, 5(6)-TAMRA-Ahx(5-24)NPY, biotin-(Ahx)_2_-NPY or [K^22^[(Ahx)_2_-biotin]Bpa^27^]NPY at Y_2_R_cysteine_deficient. EC_50_ values were determined using GraphPad Prism 5.0 nonlinear regression (curve fit), normalized to the associated NPY curves. n ≥ 2.

**
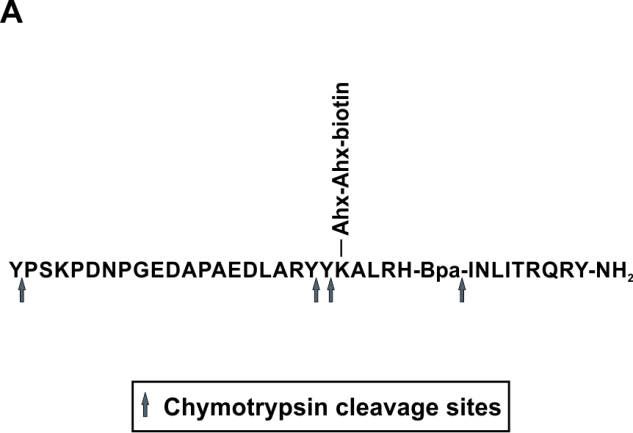

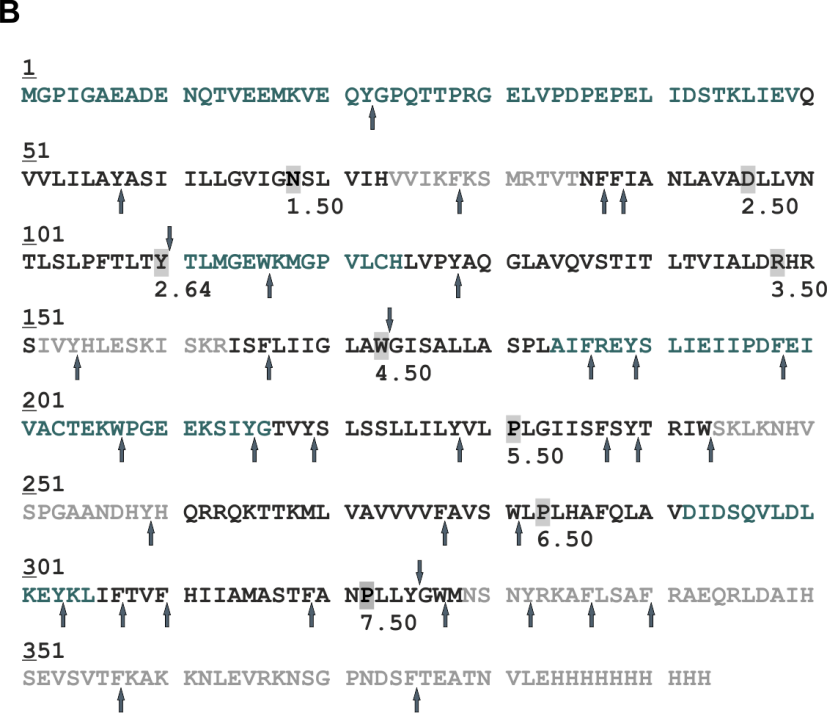
**

**Supplementary Figure S2.** Restriction sites in [K^22^[(Ahx)_2_-biotin]Bpa^27^]NPY and Y_2_R_cysteine_deficient after digestion with the endoproteinase chymotrypsin. **(A)** Amino acid sequence of [K^22^[(Ahx)_2_-biotin]Bpa^27^]NPY with highlighted restriction sites. **(B)** Amino acid sequence of Y_2_R_cysteine_deficient with a C-terminal deca-histidine-tag. N terminus and extracellular loops are highlighted in cyan, transmembrane helices in black and intracellular loops and the modified C terminus in gray. Restriction sites of chymotrypsin are indicated.

**
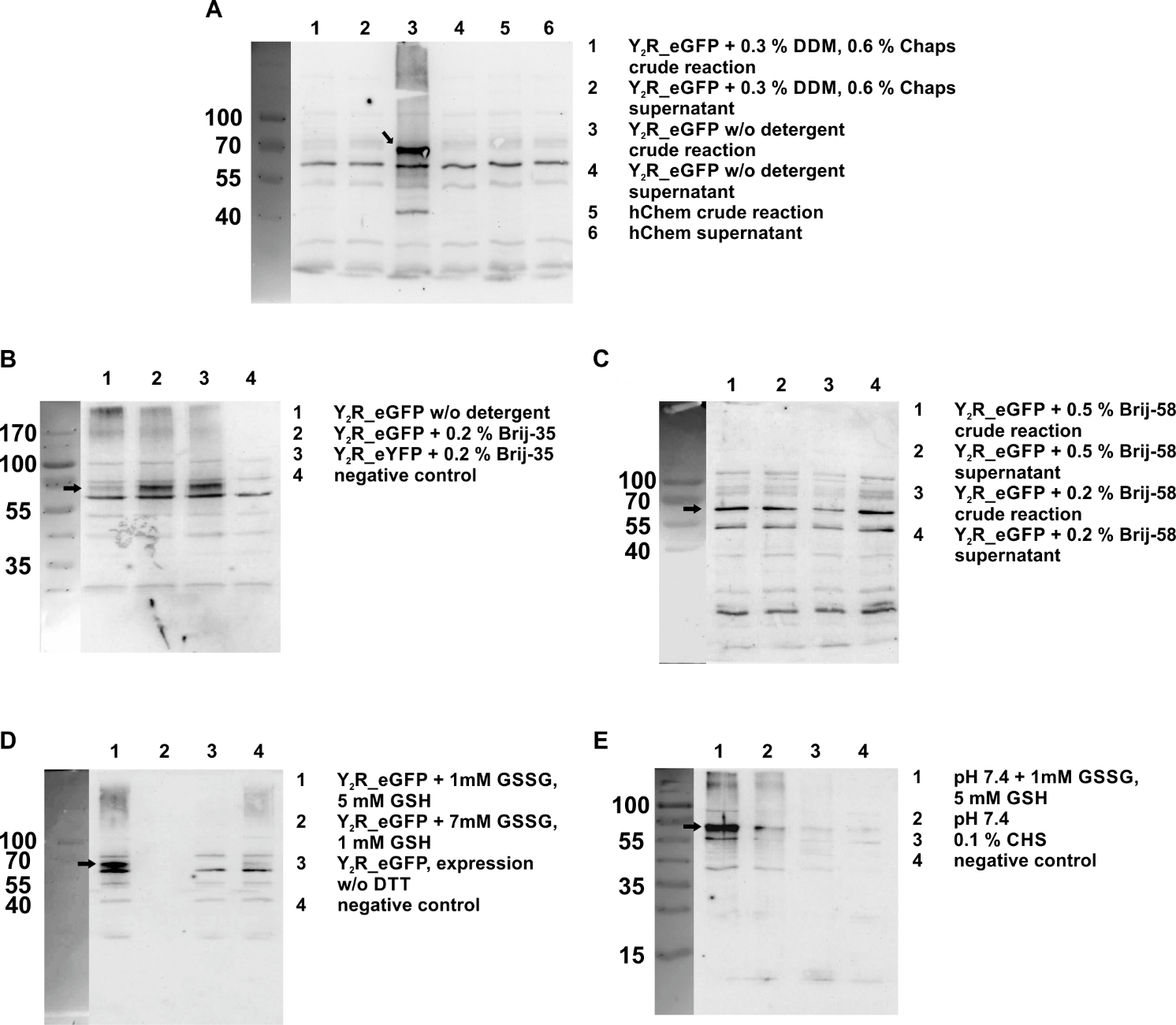
**

**Supplementary Figure S3. Western Blots of the optimization of cell-free Y_2_R_eGFP expression**. **(A)** To monitor soluble receptor expression the crude reaction (1,3,5) was centrifuged for 2 min at 13,000 rpm and the supernatant (2,4,6) was used for analysis. Y_2_R_eGFP expression was performed without detergents (1,2) or in presence of DDM and Chaps (3,4), which completely inhibit the receptor expression. As expression control, human chemerin was used, expressed without detergents (5,6). **(B)** Soluble expression of Y_2_R_eGFP (1,2) or Y_2_R_eYFP (3) without detergents (1) or in presence of Brij-35 (2,3). Nonsoluble protein was removed after expression by centrifugation. As control the RM without DNA was plotted (4). **(C)** Soluble expression of Y_2_R_eGFP in presence of 0.5 % (w/v) (1,2) or 0.2 % (w/v) Brij-58. Crude reaction and supernatant after centrifugation for 2 min at 13,000 rpm were plotted to monitor soluble receptor expression. **(D)** The Y_2_R_eGFP expression in presence of oxidized (GSSG) and reduced (GSH) glutathione was monitored by Western Blot analysis. A higher amount of GSH promotes receptor expression (1), while an increased amount of GSSG strongly inhibits it (2). A change in the redox potential during expression by omitting DTT during expression (3) completely inhibits receptor expression. The RM without DNA was used as control (4). **(E)** Buffer pH was lowered towards pH 7.4 (2) and the effect of GSH and GSSG (1) or CHS (3) addition during expression was monitored. The negative control contents the RM without DNA (4). Arrows indicate synthesized receptors. Brij-35 – polyoxyethylene(23)laurylether, Brij-58 – polyoxyethylene(20)cetyl-ether, Chaps – 3-[(3-cholamidopropyl)dimethylammonio]-1-propansulfonat, CHS – cholesteryl hemisuccinate; DDM – n-dodecyl β-D-maltoside, DTT – dithiothreitol**,** RM – reaction mix; n ≥ 2.


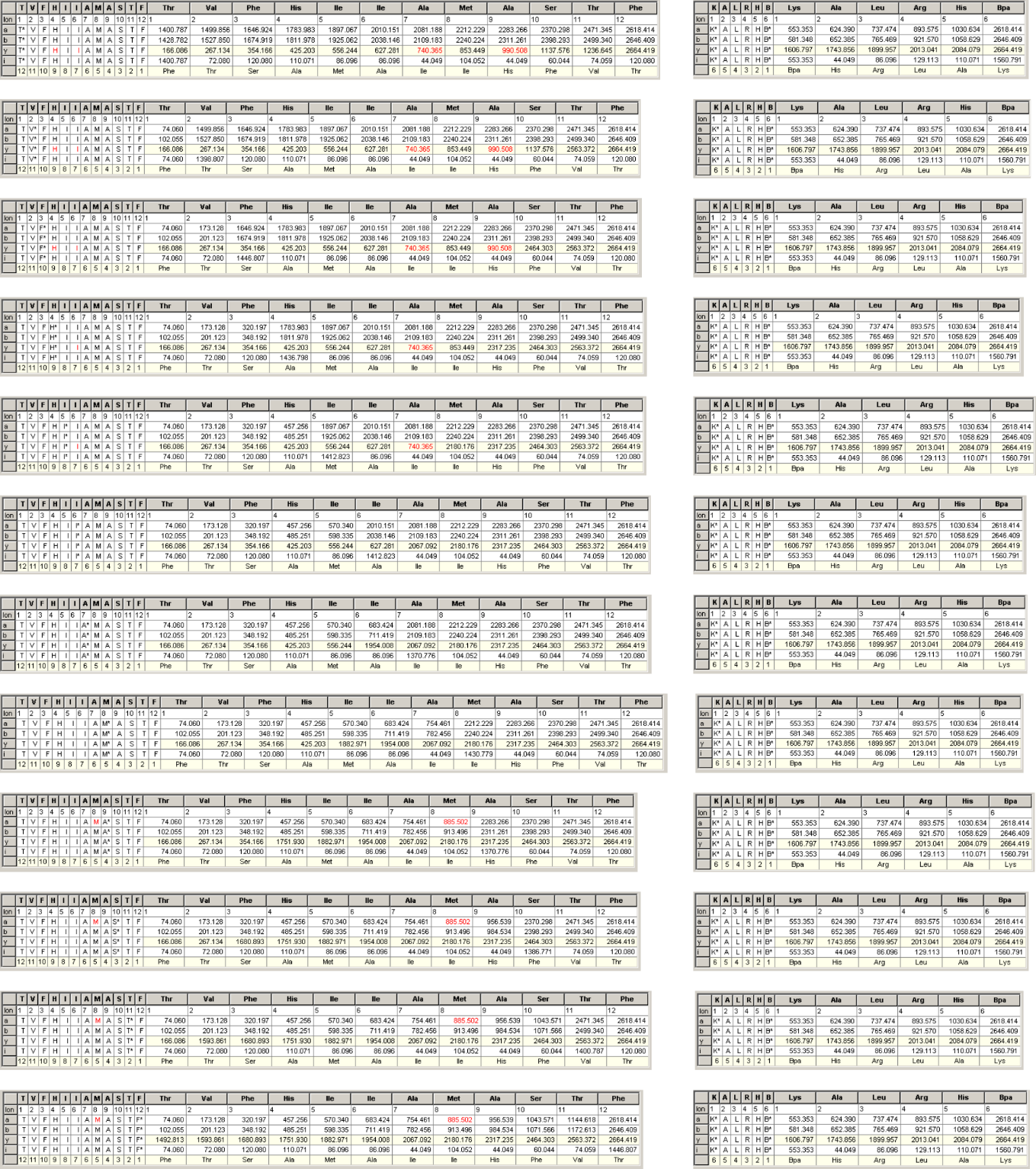


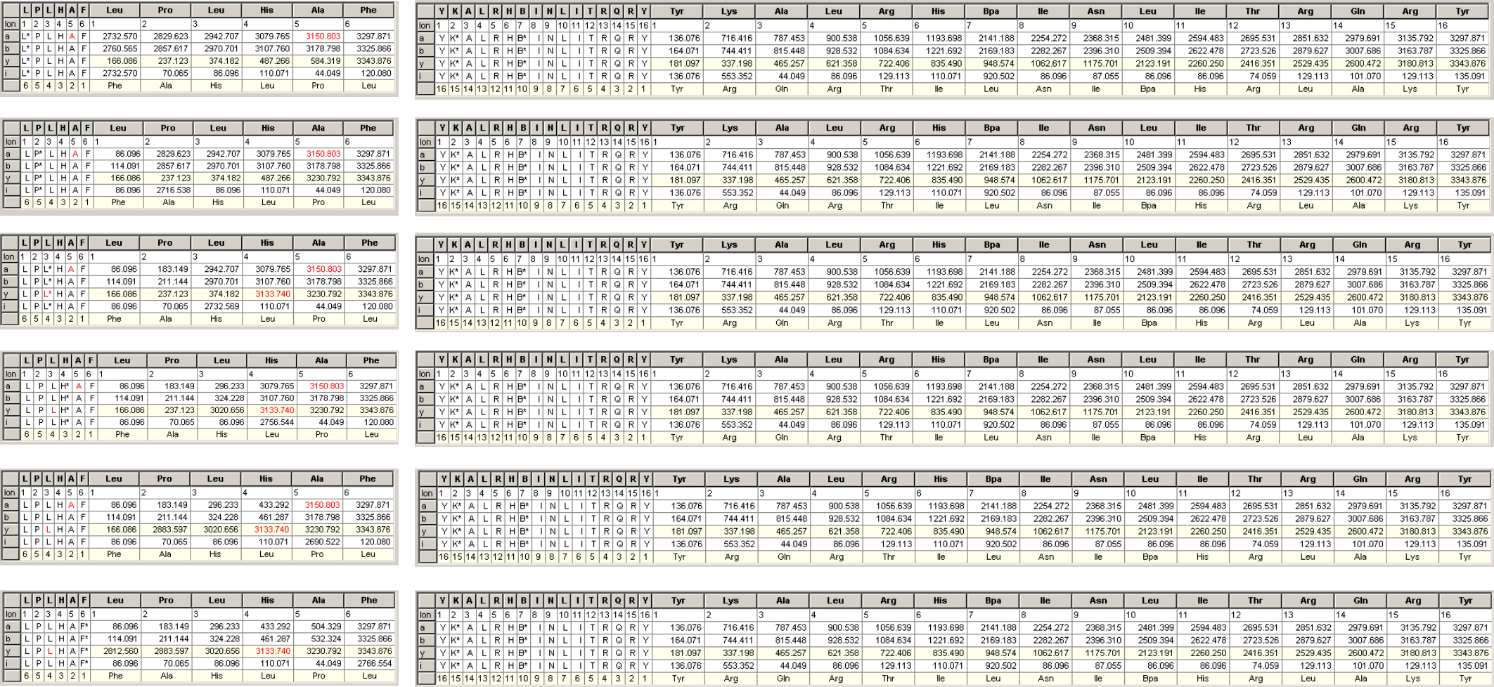


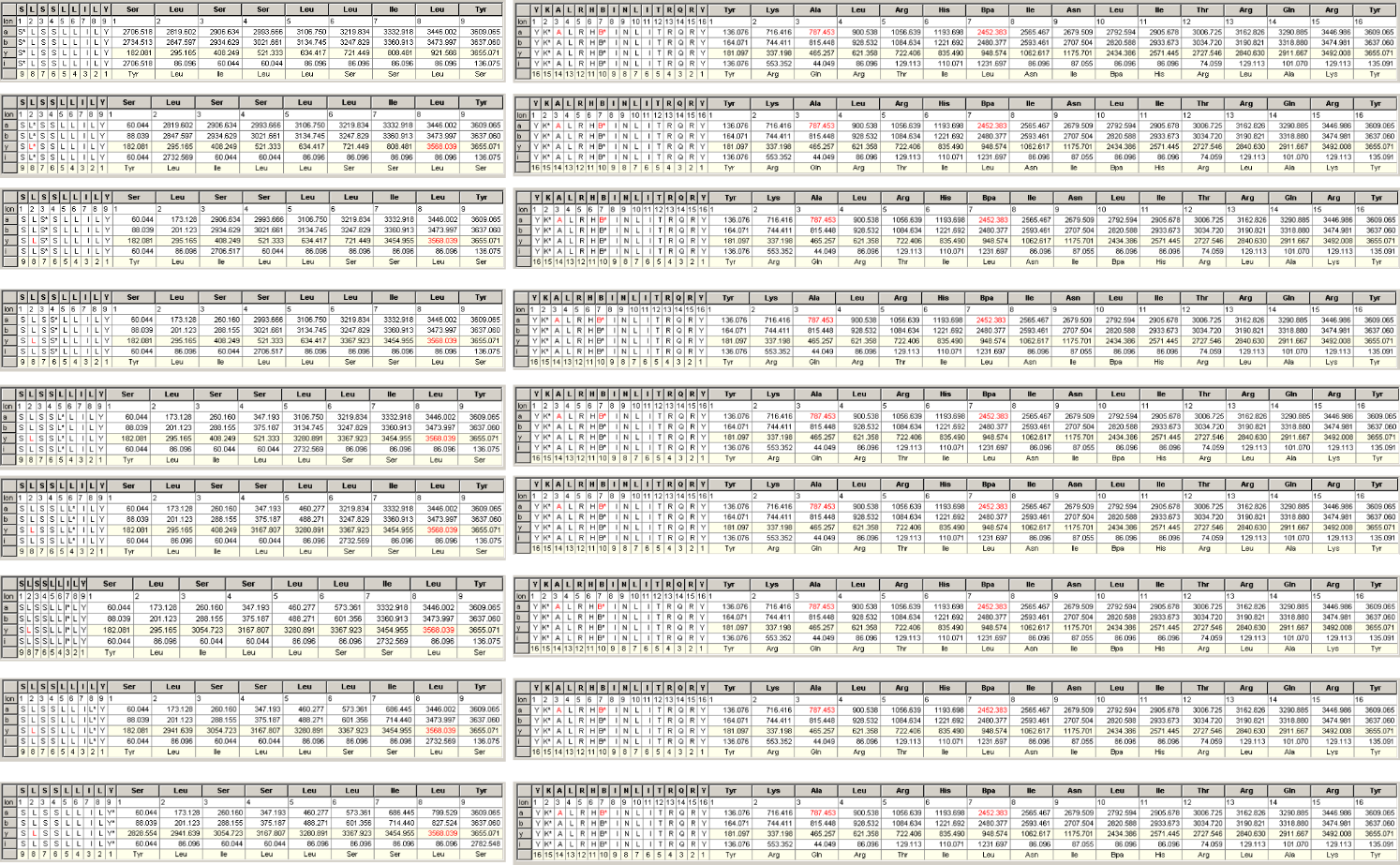


**Supplementary Figure S4:** Analysis of tandem MS/MS spectra by Biotools, allowing crosslinking to occur between Bpa at position 27 of NPY and one amino acid sequence of the identified receptor fragment at a time. (A) Parent ion 2665.5. (B) Parent ion 3345.0 (C) Parent ion 3655.1.
